# Oral Vaccination of Free-Living Badgers (*Meles meles*) with Bacille Calmette Guérin (BCG) Vaccine Confers Protection against Tuberculosis

**DOI:** 10.1371/journal.pone.0168851

**Published:** 2017-01-25

**Authors:** Eamonn Gormley, Deirdre Ní Bhuachalla, James O’Keeffe, Denise Murphy, Frank E. Aldwell, Tara Fitzsimons, Paul Stanley, Jamie A. Tratalos, Guy McGrath, Naomi Fogarty, Kevin Kenny, Simon J. More, Locksley L. McV. Messam, Leigh A. L. Corner

**Affiliations:** 1 School of Veterinary Medicine, University College Dublin (UCD), Dublin, Ireland; 2 Dept of Agriculture, Food and the Marine, Head of Wildlife Unit, Agriculture House, Dublin, Ireland; 3 Immune Solutions Ltd, Centre for Innovation, University of Otago, Dunedin, New Zealand; 4 UCD Centre for Veterinary Epidemiology and Risk Analysis (CVERA), School of Veterinary Medicine, University College Dublin (UCD), Dublin, Ireland; 5 Central Veterinary Research Laboratory, Backweston, Co. Kildare, Ireland; Public Health England, UNITED KINGDOM

## Abstract

A field trial was conducted to investigate the impact of oral vaccination of free-living badgers against natural-transmitted *Mycobacterium bovis* infection. For a period of three years badgers were captured over seven sweeps in three zones and assigned for oral vaccination with a lipid-encapsulated BCG vaccine (Liporale-BCG) or with placebo. Badgers enrolled in Zone A were administered placebo while all badgers enrolled in Zone C were vaccinated with BCG. Badgers enrolled in the middle area, Zone B, were randomly assigned 50:50 for treatment with vaccine or placebo. Treatment in each zone remained blinded until the end of the study period. The outcome of interest was incident cases of tuberculosis measured as time to seroconversion events using the BrockTB Stat-Pak lateral flow serology test, supplemented with post-mortem examination. Among the vaccinated badgers that seroconverted, the median time to seroconversion (413 days) was significantly longer (p = 0.04) when compared with non-vaccinated animals (230 days). Survival analysis (modelling time to seroconversion) revealed that there was a significant difference in the rate of seroconversion between vaccinated and non-vaccinated badgers in Zones A and C throughout the trial period (p = 0.015). For badgers enrolled during sweeps 1–2 the Vaccine Efficacy (VE) determined from hazard rate ratios was 36% (95% CI: -62%– 75%). For badgers enrolled in these zones during sweeps 3–6, the VE was 84% (95% CI: 29%– 97%). This indicated that VE increased with the level of vaccine coverage. Post-mortem examination of badgers at the end of the trial also revealed a significant difference in the proportion of animals presenting with *M*. *bovis* culture confirmed lesions in vaccinated Zone C (9%) compared with non-vaccinated Zone A (26%). These results demonstrate that oral BCG vaccination confers protection to badgers and could be used to reduce incident rates in tuberculosis-infected populations of badgers.

## Introduction

In Ireland the badger (*Meles meles*) population acts as a reservoir for *Mycobacterium bovis* infection and contributes to the persistence of tuberculosis in associated cattle populations [1]. This cross species transmission can act as a constraint to eradication of the disease from cattle. The choices for addressing tuberculosis in the badger reservoir host are often limited by conservation and social concerns, though vaccination against tuberculosis is an option that could directly facilitate the advancement of eradication in affected areas by reducing the burden of infection in wildlife [2]. The *M*. *bovis* Bacille Calmette-Guérin (BCG) Danish strain 1331 is currently the vaccine of choice for use in badgers based on the level of experience from its application in humans and domestic and wild animals. Despite questions over its efficacy in humans, there has been a concerted effort to adapt the BCG vaccine for use in a variety of animal species [3]. Programmes of research into vaccination of badgers have been undertaken in Ireland and the UK and there is clear evidence that the BCG vaccine can induce a protective immune response when delivered by a variety of routes, including oral, that limits the distribution and severity of tuberculosis following experimental challenge [4–7]. An injectable BCG vaccine for badgers was granted a license for use in the UK in 2010, and a field trial of this vaccine demonstrated that the vaccine reduced the number of *M*. *bovis* seropositive badgers by up to 74%, compared with non-vaccinated badgers, and conferred protection to non-vaccinated cubs [8, 9].

It is likely, for pragmatic reasons, that any large-scale vaccination of wildlife against tuberculosis will be based on oral vaccine delivery [10]. An edible lipid formulation that encapsulates BCG has been developed for this purpose and controlled studies have shown that it confers a high degree of protection against experimental challenge in mice [11], in captive wildlife vector species including possums, badgers, white-tailed deer [6, 12, 13] and in cattle [14]. In a field trial conducted in New Zealand, oral delivered BCG protected possums against natural disease exposure [15].

Whereas captive animal studies are the most cost effective way of examining the protective response to vaccination, such studies cannot be used to predict whether BCG will be protective in free-ranging animals subject to natural transmission or to estimate vaccine efficacy in an open population.

In this study we conducted a field trial in County Kilkenny to determine if the oral BCG vaccine has any protective effect in wild badgers exposed to natural transmission of infection. The location of the vaccine trial area was selected through a multi-criteria process, which factored in previous badger-culling history, knowledge of sett locations and estimates of local prevalence of tuberculosis in the badger population [16]. The area covered approximately 755 km^2^ characterised by low level and rich pasture land (S1 Fig). The site was divided into three zones (A, B and C), each with a different level of vaccine / placebo coverage. Treatment of badgers was double-blinded and only decoded at the end of the trial. Badgers captured in Zone A were administered placebo by the oral route, while all captured badgers in southern Zone C were vaccinated orally with BCG. Badgers captured in the middle zone (B) were randomly assigned a dose of vaccine, or placebo, on a 50:50 basis [17]. Estimates of prevalence and changing tuberculosis incidence were obtained from the measurements of serological immune responses using the Brock (TB) Stat-Pak test, supplemented with post-mortem examination [18]. In this analysis we determined the effect of oral vaccination in vaccinated badgers relative to non-vaccinated animals under conditions of natural transmission of infection.

## Materials and Methods

This study was carried out under a research licence (B100/3187) issued by the Department of Health and Children, and a clinical trials license (RL/08/06) issued by the Department of Agriculture, Food and the Marine (DAFM). Ethical approval was obtained from the University College Dublin (UCD) Animal Research Ethics Committee (AREC-P-08-26). Registered veterinarians carried out all procedures conducted on badgers under anaesthesia.

### Capture protocol

The study area, located in the south-east of Ireland (approximately 52.6° N, 7.4° W, S1 Fig) was surveyed prior to commencement and sett locations were recorded in a Geographical Information System (GIS) database. In each sweep of capturing, attempts were made to capture badgers in the vicinity of all active setts within the trial area. All setts were visited twice within each year—autumn/winter (September to February) and spring/summer (March to July). In total, seven complete sweeps of the study area were made. Sweep one commenced in September 2009 and sweep seven was completed in October 2013, a total of 1501 days (S1 Table). Both stopped wire restraints and cage traps were used to capture badgers. Standard badger capture protocol was employed during this study, and traps were set by experienced field staff to maximise the probability of capturing badgers (for example at active sett openings and along badger runs). During the trial, each active sett was captured over a 10-night period and all traps were checked daily before 12 noon [19]. All badger data gathered at each capture was recorded on a portable device and transferred weekly to a central database, using a combination of badger id (a unique code assigned to each captured badger) and date of capture as a unique identifier for each capture event. Each badger was assigned to the zone in which it was first captured and treated with vaccine or placebo at enrolment, with the number of treated animals accumulating over the course of the trial (S2 Fig).

### Preparation of oral BCG vaccine

The BCG strain Danish 1331 was grown and the oral lipid vaccine (Liporale-BCG) prepared as previously described [20]. Briefly, BCG was grown to mid-log phase in 7H9 liquid medium (Difco) supplemented with albumin-dextrose-catalase (ADC: BBL). Bacilli were harvested by centrifugation and washed twice in phosphate buffered saline (PBS) prior to storage at -70°C. The number of colony forming units (CFU) in the frozen suspension of BCG was determined by plating on Middlebrook 7H11 agar plates (Difco). The BCG was thawed and suspended in the lipid formulation, the lipid having been liquefied by warming to 37°C. The lipid-BCG suspension was homogenised by repeated inversion, transferred to 2 ml syringes and with continued gentle mixing on a rotary mixer, allowed to solidify at 4°C. The placebo without BCG was prepared in the same way. Each vaccine dose contained approx. 1 x 10^8^ CFU of BCG. Vaccine and placebo were labelled blind and only decoded at the end of the trial. All syringes were kept refrigerated until deployment.

### Anaesthesia and delivery of oral BCG vaccine

For handling, the badgers were anaesthetised with ketamine hydrochloride (10mg/kg, Vetalar^®^, Boehringer Ingelheim) and medetomidine hydrochloride (0.1 mg/kg, Domitor, Pfizer) co-administered by intramuscular injection [21]. The age of a badger was estimated based on tooth wear and body condition, and classified at capture as either juvenile (< 12 mths), adult or old. The methodology used for determination of the age of badgers is very crude. Beyond the juvenile stage it is likely to be subject to misclassification. On each occasion, the badgers were weighed and examined for signs of disease or injury, blood (5 ml) for serology was collected by jugular venepuncture. Badgers were marked with a microchip inserted subcutaneously between the shoulders and a unique number tattooed on the skin of the inguinal region. When possible, a pharyngeal swab was collected as previously described [5]. For administration, the syringes containing vaccine (1 x 10^8^ cfu) or placebo were warmed to body temperature and the lipid mixture was deposited onto the upper pharyngeal mucosa on both sides behind the most distal molar teeth. Badgers we re-vaccinated (or placebo treated) when recaptured greater than 11 months after enrolment or after a previous treatment. The viable counts of BCG were determined after preparation of vaccine, and a proportion of syringes were also submitted to the Central Veterinary Research Laboratory for culture after delivery to badgers.

### Blood sampling and immunological analysis

The BrockTB Stat-Pak (Chembio Diagnostic Systems, New York, USA) was used to test badger serum for IgM and IgG antibodies to the antigens MPB83, ESAT-6 and CFP10 [22]. The antigen binds with antibody to form a complex that is visible as a blue band in the test window. A control band in the test window acts as a quality control for each test. A positive serological result is interpreted as evidence of infection with *M*. *bovis*. The sensitivity of the Stat-Pak immunoassay has been shown to range from 35%-58% depending on the gold standard used in the analysis (culture confirmed or detection of visible lesions), and a specificity of > 97% [23, 24].

### Time to seroconversion (Stat-Pak test)

The time to seroconversion was calculated from when a seronegative badger was enrolled in the trial (i.e. first capture). It was calculated as the mid-time point between the first positive test and the last seronegative test, added to all previous days where the badger was seronegative since enrolment. For example, if a badger was captured on three occasions at one hundred day intervals and was positive to Stat-Pak at the third capture, the estimated time to seroconversion was 250 days. The survival time for seronegative /censored animals was calculated as the total number of days between enrolment and last capture. Periods of observation commenced from when a badger was enrolled in the trial [25]. The prevalence and potential level of exposure to infection was assessed by recording the proportion of badgers positive at first capture during each sweep as a function of all badgers captured during the sweep (S2 Table).

### Post-mortem examination

At the end of the trial (sweep 7) 190 badgers that had been enrolled in the study were euthanized with an intravenous overdose (10 ml) of sodium pentobarbitone and subjected to a detailed post-mortem examination. The location of the gross lesions was recorded and tissue samples were collected for culture and histopathology as previously described except that tonsils and reproductive tract tissues were not collected [5]. Tracheal swabs were also collected during the post-mortem examination. Gross lesions were confirmed by culture or where there were histological features consistent with tuberculosis. Specimens for histopathology were fixed in 10% buffered formalin, sectioned at 3*μ*m and stained with haematoxylin and eosin for tissue architecture, and by the Ziehl-Neelsen method for acid-fast organisms. Histopathological examination was carried out to detect tuberculous granulomas containing acid-fast bacilli (AFB) [26]. Animals were considered histologically positive for tuberculosis if tissues had granulomatous lesions containing AFB or had >10 typical granulomatous lesions where no AFB were observed [27].

### Mycobacterial culture and speciation

Samples of lymph node and lung were thawed overnight, placed in a stomacher bag with 10 mL of saline and homogenised for 2 minutes at 170 revolutions per minute. The homogenate was transferred to a sterile universal tube and centrifuged at 3,000 *g* for 15 minutes. The supernatant was discarded, the pellet re-suspended in 2 mL of phosphate buffered saline and 0.2 mL added to a slant of Lowenstein-Jensen medium with pyruvate and a slant of modified 7H11 medium containing pyruvate. 500ul of inoculum was added to a BACTEC MGIT 960 tube, containing OADC growth supplement and PANTA antimicrobial mixture. The remaining inoculum was frozen at -20°C. Cultures were incubated at 37°C for 12 weeks.

Growth in MGIT 960 tubes was monitored automatically while solid media was checked each week for growth. Ziehl-Neelsen staining was performed on smears prepared from MGIT cultures that signalled, and on suspect colonies from solid media. If acid-fast bacilli were recorded, a portion of the culture was heat killed and DNA extracted for testing in a multiplex real-time PCR for identification of *M*. *bovis*, *M*. *bovis* BCG and non-tuberculous mycobacteria [28, 29]. Where contamination of media was recorded, 1 ml of the unused pellet was thawed, decontaminated with 0.075% w/v cetylpyridinium chloride (CPC) and the three media re-inoculated as described. For MGIT tubes which had no indication of growth after 12 weeks, a sample of media from each of ten tubes was pooled, heat killed and DNA extracted for testing in the same PCR. If a positive signal was obtained in the PCR, individual smears were prepared for Ziehl-Neelsen staining and PCR performed on DNA extracted from heat killed culture fluid of each individual sample.

### Statistical analysis

Data from capture events were linked with the results of blood sampling and post-mortems in a Microsoft Access database (Microsoft, US), using the capture id to join the three data sets. Statistical analyses were performed using GraphPad Prism version 6.0g for Mac OS X (GraphPad Software, USA, www.graphpad.com) and SPSS version 20. Data was tested for normality using D’Agostino & Pearson omnibus normality test. Differences between groups in time to seroconversion were analysed using student t-test and non-parametric Mann-Whitney test. For time to seroconversion analyses, data for badgers were initially examined over the total trial period, and subsequently divided into two groups based on their time of enrolment in the study. Badgers enrolled during sweeps 1 and 2 were analysed separately from badgers enrolled during sweeps 3 to 6. We chose this stratified analysis to examine whether the effectiveness of the vaccine was different in badgers as a function of accumulation of vaccinated badgers over the trial period. 42% of all vaccinated badgers in Zone C were enrolled in sweeps 1–2 while the remainder was vaccinated in sweeps 3 to 6. Time to seroconversion for each sweep-specific group was analysed using the Kaplan-Meier method in SPSS version 20 with the Log Rank test used to detect differences between the survival curves for the vaccinated and non-vaccinated groups. This took account of the number and timing of seroconversion events, and the time until last follow-up for each badger which had not experienced an event i.e. has been right censored. A Cox proportional hazards model was used to estimate hazard rate ratios (HRRs) of seroconversion along with associated 95% CI for the vaccinated compared with the non-vaccinated badgers. The hazard rate (HR) of seroconversion for a badger was defined as the instantaneous rate of seroconversion at time *t* for a badger that had not seroconverted up until that time. We first tested the proportional hazard assumption by creating a vaccine status–time interaction term in the Cox model and verifying that its coefficient was not statistically significant at the 5% (i.e., p > 0.05) level. Final estimation of the hazard rate ratio (HRR) was carried out using the change in estimate procedure [30] with forward selection to choose potential confounders from among age, sex and zone seroprevalence at enrolment for each badger in the study. Prevalence was modelled as its natural logarithm to facilitate computation of model parameters. For inclusion in the final Cox model each potential confounder had to change the HRR by 10% or more upon addition to the model. The final model was of the form, *ln (HRR) = β X* where *ln* is the natural logarithm, HR = the hazard rate of seroconversion, HRR = HR_*vaccinated*_ / HR_*Non-vaccinated*_ and *β X* = a linear combination of predictor variables (i.e. vaccine status and confounders) and regression coefficients. From the HRR we estimated the Vaccine Efficacy (VE) and 95% CI using the following relationship: *VE = 1-HRR* [31].

## Results

### Population stucture and demographics

Over the course of the trial 365 badgers were enrolled and treated in non-vaccinated Zone A, 212 badgers in vaccinated / non-vaccinated Zone B, and 357 badgers were enrolled and treated in the vaccinated Zone C. Of the badgers enrolled in Zone B, 105 were vaccinated and 107 treated with placebo. The population structure in each of the three zones was similar with respect to age and sex (Table 1). Among all enrolled badgers, 501 (54%) individuals were captured once, and 433 (46%) were captured on two or more occasions. There were no significant differences (p > 0.05) in capture frequency between badgers in Zone A, Zone B and Zone C, with a recapture rate of 50%, 49% and 47% over the whole trial period, respectively. The accumulated proportion of all badgers vaccinated or placebo-treated at each sweep is shown in S2 Fig. There was no statistically significant difference in the treatment rates accumulating in each zone at each successive sweep.

**Table 1:** Distribution of badgers enrolled in vaccine field trial by age, sex and zone of capture.

Sex	Age	Zone A	Zone B	Zone C	Total
**Male**	Juvenile	63	32	62	458
	Adult	100	56	105	
	Old	17	9	15	
	Total	179	97	182	
**Female**	Juvenile	73	42	62	476
	Adult	85	58	80	
	Old	28	15	33	
	Total	186	115	175	**934**

### Time to seroconversion among seroconverting badgers

**Fig 1:**
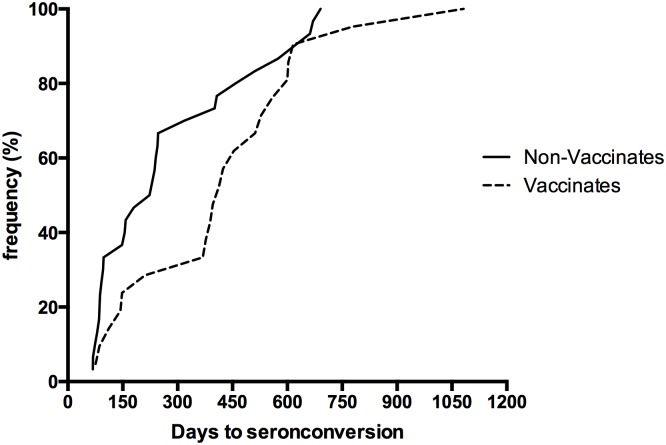
Time (days) to seroconversion for all badgers that seroconverted during the trial: non-vaccinated (N = 30), vaccinated (N = 21).

Among the non-vaccinated and vaccinated badgers across all three treatment zones there were 30 and 21 seroconversion events respectively recorded during the trial period. The frequency of seroconversion events as a function of the estimated number of days to seroconversion is shown in Fig 1. For non-vaccinated badgers, the median time (S_50_) to seroconversion (time taken for 50% of non-vaccinated badgers to seroconvert) was 230 days (95% CI: 98–318 days), which was shorter than the median time to seroconversion for the vaccinated badgers (S_50_ = 413 days: 95% CI: 211–559 days). The difference in median time to seroconversion between non-vaccinated and vaccinated badgers was 183 days (p = 0.04, 95% CI: = 1–301 days), which resulted in a higher proportion of non-vaccinated badgers seroconverting compared with vaccinated badgers during a defined time period. For example, by 420 days post-enrolment 75% of all non-vaccinated badgers that would eventually seroconvert had seroconverted while it took an additional 160 days (580 days in total) for the equivalent proportion of vaccinated badgers to seroconvert.

### Kaplan-Meier survival analysis

**Fig 2:**
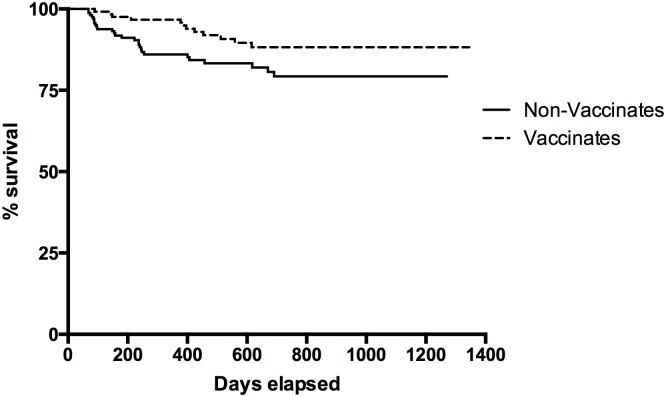
Kaplan-Meier survival curves (time to seroconversion) for non-vaccinated and vaccinated badgers during the whole period of study.

We then compared the seroconversion rates among the 100% non-vaccinated population of badgers in Zone A with the 100% vaccinated population in Zone C, and independently compared the 50:50 vaccinated and non-vaccinated populations in Zone B. Over the course of the trial there was 27 seroconversion events recorded among the non-vaccinated badgers (Zone A) and 12 among the vaccinated badgers (Zone C). Kaplan Meier analysis was used to estimate the survival time to seroconversion of all badgers in both populations during the trial period. There was a significant difference (Log Rank, p = 0.015) in the survival times to seroconversion between the vaccinated and non-vaccinated badgers (Fig 2). The mean time to seroconversion in the non-vaccinated badgers, 1,069 days (95% CI: 1,000–1,139) was substantially shorter than the time to seroconversion for vaccinated badgers, 1,233 days (95% CI: 1,174–1,294). There were also differences in the proportions of animals seroconverting during the study period. For example, at 300 days following enrolment of badgers (during any sweep) 14.0% of non-vaccinated badgers had seroconverted compared to only 2.0% of the vaccinates (7-fold difference). However, by 700 days post-enrolment, 21.0% of non-vaccinated badgers had seroconverted compared with 11.0% of vaccinated badgers (2-fold difference).

In Zone B, there were only 12 seroconversion events recorded throughout the trial period, 9 among the vaccinated badgers and 3 in non-vaccinated badgers. Although the mean time to seroconversion was shorter in the vaccinated badgers (929 days, 95% CI: 778–1081) compared with non-vaccinated animals (1182 days, 95% CI: 1016–1347 days), there was no significant difference in survival time to seroconversion between these non-vaccinated and vaccinated populations of badgers (Log Rank, p = 0.12).

### Hazard rate and vaccine efficacy

Because the badgers were enrolled at different times over the whole trial period, we investigated the rate of seroconversion in each zone as a function of time of enrolment from the beginning of the trial. We performed a stratified analysis for badgers enrolled in sweeps 1–2 and sweeps 3–6. For those enrolled in sweep 1–2, 12% among the non-vaccinated and 10% of vaccinated badgers seroconverted during the trial (Log rank test: p = 0.49). For those badgers enrolled in sweep 3–6, 22% of non-vaccinated and 5% of vaccinated badgers seroconverted (Log rank test p = 0.005). In Zone B, there was no statistical difference in the proportions of seroconverting badgers in the stratified analysis: 8 of the 12 seroconversion events occurred in badgers enrolled during sweeps 1–2 of the study (5 vaccinated and 3 non-vaccinated badgers), with the remainder enrolled in sweeps 3–6.

**Fig 3:**
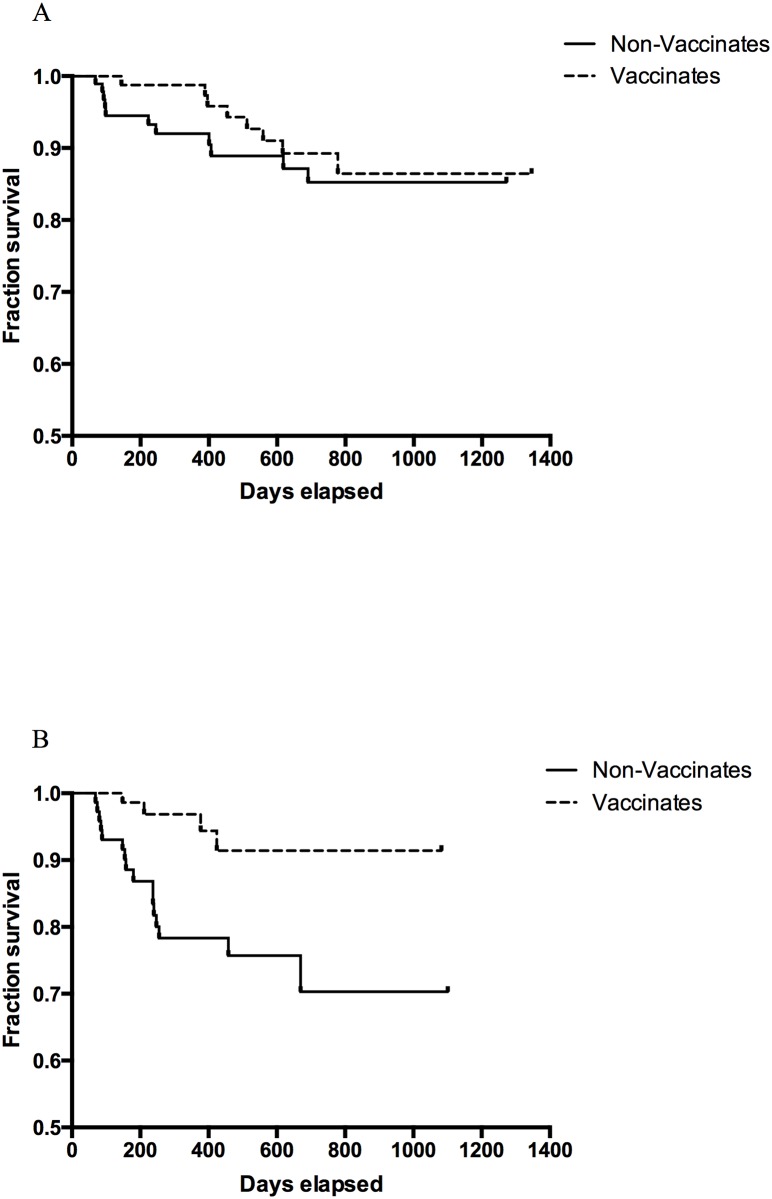
Kaplan-Meier survival curves (time to seroconversion) for non-vaccinated and vaccinated badgers enrolled in sweeps 1–2 (A) and in sweeps 3–6 (B).

For badgers enrolled in Zone C and Zone A during sweeps 1 and 2 (Fig 3A) the mean survival time for vaccinated and non-vaccinated badgers was 1233 days (95% CI: 1160–1306) and 1131 days (95% CI: 1053–1208 days) respectively (Log rank test: p = 0.49). Adjusting for age and prevalence, the HR of seroconversion for vaccinated badgers was lower than the HR of seroconversion among non-vaccinated badgers (HRR = 0.64; 95% CI: 0.25–1.62). This was equivalent to VE = 36% (95% CI: -62%– 75%).

For badgers enrolled in sweeps 3 to 6, (Fig 3B) the mean survival time was 1016 days (95% CI: 955–1079 days) and 858 days (95% CI: 754–962 days) for vaccinated and non-vaccinated badgers respectively (Log rank test p = 0.005). Adjusting for prevalence the HR of seroconversion for vaccinated badgers was 0.16 (95% CI 0.03–0.71) times the HRR of seroconversion for non-vaccinated badgers, which was equivalent to VE = 84% (95% CI: 29%– 97%).

### Post-mortem analysis of badgers

At the end of the trial (sweep 7) a cross section of the enrolled badger population (N = 181 badgers) was removed from the trial area. There was a significantly lower number of animals (p = 0.01) with culture confirmed gross lesions among the vaccinated badgers in Zone C (6/64, 9%) compared with non-vaccinated badgers in Zone A (20/77, 26%). In Zone B, there was no significant reduction (p > 0.05) in the lesion rate among vaccinates (1/9, 11%) compared with non-vaccinates (6/40, 15%). The examination of histological lesions increased the sensitivity of detection of infected animals with 14% of animals among the vaccinated badgers in Zone C presenting with histological lesions compared with 39% in the non-vaccinated group in Zone A. The proportion of badgers with gross lesions compared to histological lesions was the same in both vaccinated and non-vaccinated badgers (2:3). In Zone B, 20% of non-vaccinated badgers presented with histological lesions, whereas 44% of vaccinated badgers had evidence of histological lesions.

In assessing the performance of the Stat- Pak in these populations, for 27 badgers with confirmed lesions the serology test sensitivity was estimated to be 62% (Positive Predictive Value = 94%, 95% CI = 71% - 100%). The test sensitivity, based on histology and /or culture confirmation of infection (including visible and non-visible lesioned badgers), was estimated to be 35% (Infection prevalence = 38%, Positive Predictive Value = 78%, 95% CI = 61% - 90%) for all animals infected with *M*. *bovis*.

## Discussion

Field trials are an important element of vaccine development as they facilitate the measurement of protective immunity under conditions of natural levels of transmission [31]. This overcomes a major limitation of experimental challenge models where the objective is to achieve a uniform level of infection using relatively high challenge doses designed to generate progressive infection in all animals, including vaccinates. In this field study we have estimated the VE of oral BCG vaccination in a free-living population of badgers relative to non-vaccinated badgers. Our strategy to estimate VE was based on measuring time to seroconversion events using a structure of repeated test measures on longitudinal data [31]. There are currently no immunological biomarkers for badgers that correlate with protective immunity generated by BCG vaccination. As such, the detection of serological responses associated with advanced infection serves only as a proxy for loss of protection in the vaccinated population. In several experimental vaccine/challenge studies we have previously demonstrated high levels of protection when the BCG was delivered to badgers by a variety of routes, including oral inoculation [4–6]. The findings in this study of a significant protective effect of oral BCG vaccination in badgers under natural conditions are also consistent with the results of other oral vaccination studies in cattle [14], deer [13] and wild boar [32, 33]. The results of a field trial conducted in brushtail possums in New Zealand under conditions of natural transmission, using the same lipid encapsulated vaccine employed in this study, also found a VE exceeding 90% [15].

In our study we found a reduction in the overall hazard risk of seroconversion in vaccinated animals in Zone C relative to non-vaccinated badgers in Zone A, and where seroconversion occurred, a significant time delay to a seropositive test result in vaccinated badgers compared with non-vaccinates across all three treatment zones.

The delayed time to seroconversion has previously been observed in experimental vaccine/challenge studies where vaccine protection in badgers generated by two different delivery routes, subcutaneous route and mucosal route, was expressed as a significant delay in time to seroconversion after endo-bronchial *M*. *bovis* challenge [34]. In that study the change in time to seroconversion also correlated with the progression and severity levels of the experimental infection. It was proposed that the time to seroconversion might therefore represent a reliable surrogate for vaccine-mediated protection against disease progression. This proposition is supported by the current study under conditions of natural transmission where multiple factors affecting transmission might vary and the challenge dose is predicted to be lower than in experimental infections.

Because of their geographical separation we were able to assess the direct effect of vaccination when comparing Zone A with Zone C, confident that there was limited mixing between the two populations. In contrast, Zone B was spatially linked to Zones A and C increasing the likelihood of mixing of badgers along the boundaries, which could act as a confounder if the results were combined. In addition, due to a low number of seroconversion events occurring in Zone B badgers the survival analysis as described for Zones A and C was not suitable to determine the impact of vaccination on this population. The results of survival analysis showed that the rate of seroconversion was significantly higher in the non-vaccinated population in Zone A compared with the vaccinated badgers in Zone C. At 300 days post-enrolment of badgers enrolled at any time point during the trial the proportion of seroconversions was 7-fold greater among the non-vaccinated badgers compared with vaccinates. This difference was reduced to 2-fold by day 700 following enrolment. This could indicate a waning immunity in a proportion of vaccinated badgers over the time period of observation. However, other explanations should be considered. Because of the misclassification of the infection status of Stat-Pak test false-negative badgers at the time of vaccination (because of imperfect test sensitivity), it is highly probable that some of the vaccinated *M*. *bovis* infected /seronegative badgers seroconverted during the trial. We believe the likely effect of this misclassification would be to attenuate the observed positive effects of the vaccine particularly if the principle effect of the vaccine is to protect against infection.

As time of enrolment was considered as a proxy for vaccination coverage we conducted a stratified analysis of badgers enrolled in sweeps 1–2 and sweeps 3–6 to investigate if VE changed with increased vaccine coverage. The direct effect of vaccination on the rate of seroconversion was evident (although statistically non-significant) in badgers enrolled during sweeps 1–2 with a VE = 36%, when 42% of all trial badgers enrolled in Zone C were vaccinated. It is probable that during these early sweeps the population in Zone C would have contained a higher proportion of susceptible animals and animals misclassified by the serology test (false negatives) compared with subsequent sweeps. The age and background prevalence at enrolment were confounders, although exclusion of these covariates from the model did not significantly change VE. In contrast, vaccination of badgers enrolled in sweeps 3–6 resulted in a statistically significant VE = 84% (adjusted for seroprevalence at time of enrolment) indicating that protection of the vaccinated population increased with vaccine coverage. These results suggested an interaction between the time of enrolment (relative to the beginning of the trial) and the VE. In other words, the effect of the vaccine as expressed by VE was modified by time of enrolment leading to an increase in VE as the level of coverage increased. An average VE calculated for the whole time period would not be accurate, as it would obscure the vaccine coverage dependent interaction, which of itself is important in understanding the causal mechanism behind the VE. Also, an average VE would produce an estimate midway between the two values but not applicable to any of the time periods that were actually investigated. As the vaccine coverage increased in successive sweeps, the proportion of protected badgers also increased and served to reduce the proportion of susceptible animals in the population. In addition, the nature of protection conferred by vaccination may have been important for the decline in incidence if a vaccinated badger became infected and altered the progression of post-infection disease. If the vaccinated badgers were protected from infection or there was delayed seroconversion then these effects may have resulted in reduced infectiousness and risk of transmission (direct effect), and as the proportion of vaccinated badgers increased, this may have reduced the rate of infection in the remainder of the, as yet, non-vaccinated population (indirect effect).

The results of the post-mortem examination of badgers euthanized during sweep 7 are also consistent with a vaccine—mediated decline in disease in Zone C. When expressed as culture-confirmed lesions and histological infection, disease was much more frequent at the end of the study in Zone A compared with Zone C. The detection of lesions by histological examination is more sensitive and detects a greater range of stages of disease, whereas visual inspection for gross lesions detects only the larger lesions resulting from progression of infection. Gross lesions are more likely to develop in the more susceptible animals and also in animals with longer duration of infection. Challenge dose and route of infection may also be important. Given the short duration of the trial, the probability of infected animals presenting with gross lesions is likely highest in those animals enrolled earlier in the trial. It is in this context that the delay in time to seroconversion in badgers in Zone C relative to Zone A is consistent with a corresponding decrease in disease levels among the vaccinates.

In several studies, the sensitivity of the Stat-Pak immunoassay has been shown to range from 35%-58% with a specificity of > 97%, depending on the cohorts of animals used in the analysis [23, 24]. Based on the post-mortem data from the badgers euthanized at the end of the trial, the Stat-Pak performance was also consistent with published values, with sensitivities of 62% and 35% for *M*. *bovis* infected badgers, with and without lesions, respectively. The positive predictive value of the test for animals with confirmed lesions indicates a high probability of advanced tuberculosis infection given a Stat-Pak test positive result.

The post-mortem results also suggested a decrease in overt disease in the vaccinated animals in Zone B compared with the non-vaccinates, though the level of histological infection was higher in the small number of vaccinates examined. All of the badgers euthanized at the end of the trial are being subjected to a detailed bacteriological examination where isolation and distribution of *M*. *bovis* in infected animals will be used to measure the direct effect of vaccine (and re-vaccination) at a more refined level, and to take account of any indirect vaccine effect on the 50% of animals that received placebo in Zone B. The results will be reported in due course.

The combination of pathology and serology data indicated a relatively high burden of tuberculosis infection in all populations throughout the trial. By the end of the study the background seroprevalence had increased in the non-vaccinated Zone A but remained comparatively stable in the vaccinated Zone C and in Zone B. Over the relatively short timeframe of the study, many seroconverted badgers were recaptured in follow-up sweeps, and others were captured on a single occasion. It is only when the loss of seroconverted badgers from the population, due to migration or death, exceeds the seroconversion rate that the reduced incidence rate in the vaccinated area would lead to lower prevalence. This would be expected to eventually occur as the proportion of vaccinated badgers increases. A variety of vaccine strategies can be conceived to optimize the impact of vaccination on reducing incidence rates of infection. The choice of any particular strategy is likely to be influenced by the burden of disease in the targeted population, the long-term cost / benefit of deployment of vaccine and a host of other epidemiological parameters that will require detailed consideration [10]. A key objective of vaccination of badgers is to reduce the infection transmission rates to cattle and any cost / benefit analysis will need to factor in the benefit accruing from a reduction in the rate of herds breaking down with tuberculosis. The vaccine trial described here was not designed to test the impact of badger vaccination on cattle reactor rates, and any effect on cattle would need to be measured for many more years. In any event, the removal of a significant number of badgers at the end of the trial is also likely to have changed the epidemiology of the disease, which would confound any ongoing influence of vaccination on transmission of infection to cattle. Given the high burden of disease in the trial area it is likely that vaccination alone, even at high coverage levels would require many years to eradicate tuberculosis from a population of infected badgers [35]. Simulation modeling studies have been conducted to assess the impact of mixed control strategies, including vaccination and culling, on levels of bovine tuberculosis [36, 37]. Over periods of 10–20 years culling appeared most effective as a single strategy, however this approach posed a high risk of local extinction of badgers. The analysis showed that mixed strategies incorporating culling (to reduce density and/or remove infected badgers) and vaccination can also be effective while reducing the long-term threat to population viability and survival, and may be the most cost efficient way of eradicating the disease [36].

The results of this trial have established for the first time that oral vaccination of free-living badgers can reduce the incidence of tuberculosis in free-ranging badger populations. Prior to wide-scale deployment of an oral vaccine containing live BCG it will need to be licensed as a veterinary medicine and receive authorization for use from regulatory agencies. This should pave the way for development of mixed control strategies to incorporate vaccination into programmes to eradicate tuberculosis from badgers and cattle.

## Supporting Information

S1 Fig Map location of vaccine trial site in Co. Kilkenny. (JPG)

S2 Fig Cumulative frequency of enrolment of vaccinated or non-vaccinated treated badgers in all zones over the course of the trial. There were no statistically significant differences between the groups (P > 0.05).
(TIFF)

S1 Table Sweep length intervals 2009–2013: There was no captures conducted in July (apart from sweep 2) and August. Duration of trial = 1,501 days.
(DOCX)

S2 Table Seroprevalence estimates in Zones A, B and C expressed as moving average at successive sweeps. A seroprevalent case was defined as an animal seropositive in the StatPak test at enrolment.
(DOCX)
