# Supplementary figures and images for: Oral Vaccination of Free-Living Badgers (Meles meles) with Bacille Calmette Guérin (BCG) Vaccine Confers Protection against Tuberculosis
Source: PLoS One. 2017 Jan 25;12(1):e0168851. doi: 10.1371/journal.pone.0168851 (PMC5266210; doi:10.1371/journal.pone.0168851)

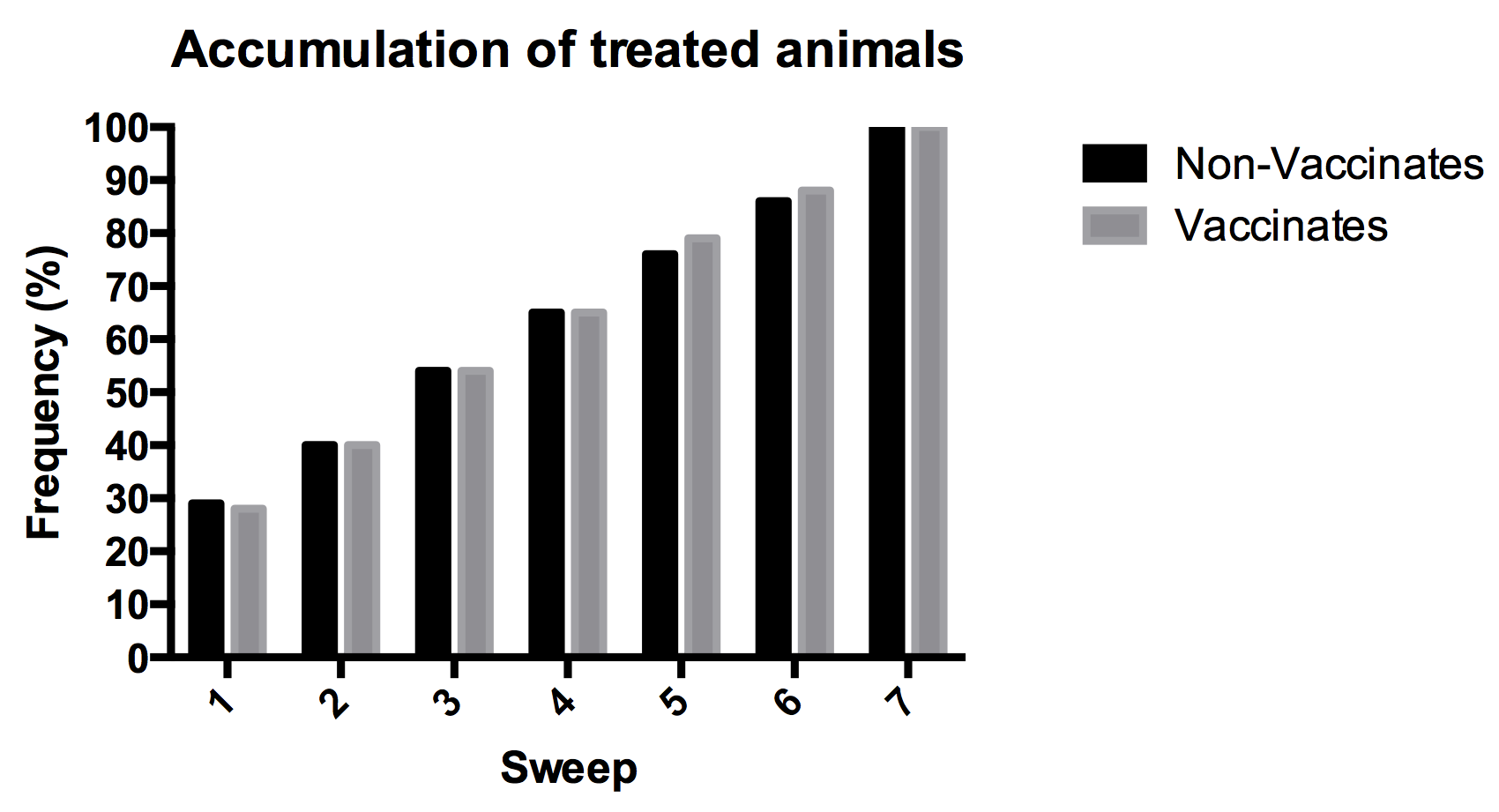

Supplement: S2 Fig — There were no statistically significant differences between the groups (P > 0.05). (TIFF) [file pone.0168851.s002.tiff]
