# Supplementary material for: Oral Vaccination of Free-Living Badgers (Meles meles) with Bacille Calmette Guérin (BCG) Vaccine Confers Protection against Tuberculosis
Source: PLoS One. 2017 Jan 25;12(1):e0168851. doi: 10.1371/journal.pone.0168851 (PMC5266210; doi:10.1371/journal.pone.0168851)
Supplement: S1 Table — Duration of trial = 1,501 days. (DOCX) [file pone.0168851.s003.docx]

|  | **Start** | **End** | **No. days** |
| --- | --- | --- | --- |
| **sweep 1** | 01/09/2009 | 19/02/2010 | 172 |
| **sweep 2** | 02/03/2010 | 16/07/2010 | 137 |
| **sweep 3** | 01/09/2010 | 04/02/2011 | 157 |
| **sweep 4** | 08/02/2011 | 29/06/2011 | 142 |
| **sweep 5** | 08/09/2011 | 27/01/2012 | 142 |
| **sweep 6** | 31/01/2012 | 22/06/2012 | 144 |
| **sweep 7** | 18/09/2012 | 11/10/2013 | 386 |
