# Supplementary material for: Oral Vaccination of Free-Living Badgers (Meles meles) with Bacille Calmette Guérin (BCG) Vaccine Confers Protection against Tuberculosis
Source: PLoS One. 2017 Jan 25;12(1):e0168851. doi: 10.1371/journal.pone.0168851 (PMC5266210; doi:10.1371/journal.pone.0168851)
Supplement: S2 Table — A seroprevalent case was defined as an animal seropositive in the StatPak test at enrolment. (DOCX) [file pone.0168851.s004.docx]

|  | Sweep 1-2 | Sweep 2-3 | Sweep 3-4 | Sweep 4-5 | Sweep 5-6 | Sweep 6-7 |
| --- | --- | --- | --- | --- | --- | --- |
| Zone A | 0.086 | 0.053 | 0.014 | 0.2 | 0.25 | 0.27 |
| Zone B | 0.095 | 0.1 | 0.13 | 0.16 | 0.16 | 0.16 |
| Zone C | 0.056 | 0.055 | 0.075 | 0.05 | 0.065 | 0.075 |
